# *PAX9* Polymorphisms and susceptibility with sporadic tooth agenesis in Turkish populations: a case-control study

**DOI:** 10.1186/1471-2164-14-733

**Published:** 2013-10-26

**Authors:** Eren Isman, Suleyman Nergiz, Hasan Acar, Zafer Sari

**Affiliations:** 1 Department of Orthodontics, Faculty of Dentistry, Gaziantep University, Gaziantep 27310, Turkey; 2 Department of Biochemistry, Selcuklu Faculty of Medicine, Selcuk University, Konya, Turkey; 3 Department of Medical Genetics, Faculty of Medicine, Selcuk University, Konya, Turkey; 4 Department of Orthodontics, Faculty of Dentistry, Akdeniz University, Antalya, Turkey

**Keywords:** *PAX9*, Hypodontia, Tooth agenesis, Oral genetics, Polymorphism, RFLP

## Abstract

**Background:**

Hypodontia, the congenital absence of one or a few teeth is one of the most common alterations of the human dentition. Familial hypodontia is caused by mutations in *PAX9*, Msx1 and Axin2 genes. Limited numbers of studies are present to show etiological factors beyond this anomaly in Turkish community belonging to Caucasian racial family. The purpose of this study is to investigate the relationships between the two different single nucleotide polymorphisms that are G-1031A and T-912C with hypodontia in Caucasians.

200 individuals having hypodontia and 114 normal individuals having all 32 teeth present were selected for the study. Blood samples were collected from each individual and DNA was extracted. To determine the polymorphisms, PCR-RFLP method was used.

**Results:**

The outcomes suggest that the individuals having AC haplotype carry less risk in having hypodontia compared with the rest of the haplotype groups (OR = 3.88; CI = 95%; p = 0.001). The ratio of GT haplotype is less in the hypodontia group meaning that the GT carriers are in risk group in terms of hypodontia risk.

**Conclusion:**

These results indicate that polymorphisms in the promoter region of *PAX9* gene may have an influence on the transcriptional factors and activity of this gene and are associated with hypodontia in Caucasian individuals.

## Background

Teeth are an important part of the digestive system. There are more than 200 genes in the control mechanism of tooth development [1]. The majority of those genes are integrated with conserved signaling pathways mediating cellular activity, in particular between epithelial and mesenchymal tissues [2]. If a problem occurs related to these genes, the pathway may be blocked and tooth, which is the outcome of the signaling cascade, may not be acquired.

Congenital tooth agenesis can be found in many patients during dental practices, and the prevalence of tooth agenesis can be between 0.027% [3] and 11.3% [4]. The most frequent missing teeth are third molars, with a prevalence of 15–20% of patients [5]. The etiology of congenital tooth agenesis is complex and is not clearly known, but generally it can be attributed to genetic and environmental factors [6]. Developmental anomalies, endocrine tissue disorders, oral cavity originated pathologies, trauma to head and neck region, medical therapies, early received radiotherapy, high fever, wrong nutrition in pregnancy, and rubella-type diseases can be considered as environmental factors [7-10].

Genetically, tooth agenesis can be either syndromic or non-syndromic. The most recent studies show that *MSX1*[11], *PAX9*[12,13], *AXIN2*[14], *TGFA*[15], *IRF6*[16], *MMP1* and *MMP20*[17], *AXIN2*[18] and *FGF3*[19] genes are related to sporadic type tooth agenesis. Peters [20] declared that mice having a mutated *PAX9* gene can show craniofacial abnormalities, extremity anomalies, and teeth developmental problems, such as halted tooth development during the bud phase. He added that all the members of a family having a frame-shift mutated *PAX9* gene had missing permanent first molars, while all deciduous teeth were healthy and properly positioned.

The expression of *PAX9* also modulates the expression of other crucial developmental regulatory genes, such as *MSX1* and Bmp4, and antagonistic interactions between *FGF8* and *BMP2* or *BMP4* control the initiation of *PAX9* expression in mandibular mesenchyme [21].

Genetic polymorphisms often show ethnic variation. Alleles, which are elements of a number of alternative forms of the same gene or same genetic locus (the specific location of a gene or DNA sequence on a chromosome) for a character producing different effects, may play role in this issue. For example, the frequency of the variant allele [ G (guanine) ] allele of rs2073244 (adenine) A > G was 25% in a Brazilian population [22] but 48% in a Chinese population [23]. Some studies have noted the prevalence of congenital missing teeth in a Turkish population [24], and limited studies could be found about the genetic background of hypodontia in a Turkish community which belongs to Caucasians [16-19]. As studies of different ethnic populations are needed to ascertain the association between genetic polymorphisms of *PAX9* and sporadic tooth agenesis [23], the purpose of this study was to investigate the relationship between two different single nucleotide polymorphisms (G-1031A and T-912C) (NCBI ref SNP ID: rs 2073247 and rs 2073244) of the *PAX9* gene promoter region and hypodontia in a Turkish population which is a member of Caucasian racial family.

## Results and discussion

### Dentition profiles and genotypes of cases

The permanent dentition profile of the individuals suffering from different types of tooth agenesis and their genotypes of the *PAX9* -1031 and −912 promoter regions are shown in Table 1.

**Table 1:** Dentition profiles and genotypes of first twenty five patients out of 200 individuals suffering tooth agenesis (dark background refers to maxillary teeth and white background refers to mandibular teeth)

**G-1031A**	**C-912 T**		**Patient.#**	**8R**	**7R**	**6R**	**5R**	**4R**	**3R**	**2R**	**1R**	**1L**	**2L**	**3L**	**4L**	**5L**	**6L**	**7L**	**8L**
GG	**CC**	Upper	1							X									
		Lower																	
GA	**CT**	Upper	2							X			X						
		Lower																	
GA	**CT**	Upper	3													X			
		Lower					X									X			
GA	**CT**	Upper	4																
		Lower														X			
GA	**CT**	Upper	5							X									
		Lower					X												
GG	**CC**	Upper	6							X			X						
		Lower					X						X						
GG	**CC**	Upper	7							X									
		Lower																	
GA	**CT**	Upper	8							X									
		Lower					X									X			
GA	**CC**	Upper	9																
		Lower					X												
GA	**CT**	Upper	10																
		Lower					X									X			
GA	**CT**	Upper	11				X	X	X	X			X	X					
		Lower					X			X	X	X	X	X		X			
GA	**CT**	Upper	12							X									
		Lower																	
GA	**CT**	Upper	13							X			X						
		Lower								X			X						
GG	**CC**	Upper	14							X			X						
		Lower																	
GG	**CC**	Upper	15							X			X						
		Lower																	
GA	**CT**	Upper	16																
		Lower					X									X			
GG	**CC**	Upper	17				X			X			X			X			
		Lower					X									X			
GG	**CC**	Upper	18																
		Lower					X												
GA	**CT**	Upper	19												X				
		Lower																	
GG	**CC**	Upper	20							X			X						
		Lower																	
GG	**CT**	Upper	21							X			X						
		Lower																	
GG	**CC**	Upper	22																
		Lower					X									X			
GA	**CT**	Upper	23																
		Lower				X					X	X				X			
GA	**CT**	Upper	24							X			X						
		Lower					X									X			
GG	**CC**	Upper	25							X			X						
		Lower																	

( X ) refers to missing teeth.

### Product of PCR–restriction fragment length polymorphism gel electrophoresis

**Figure 1:**
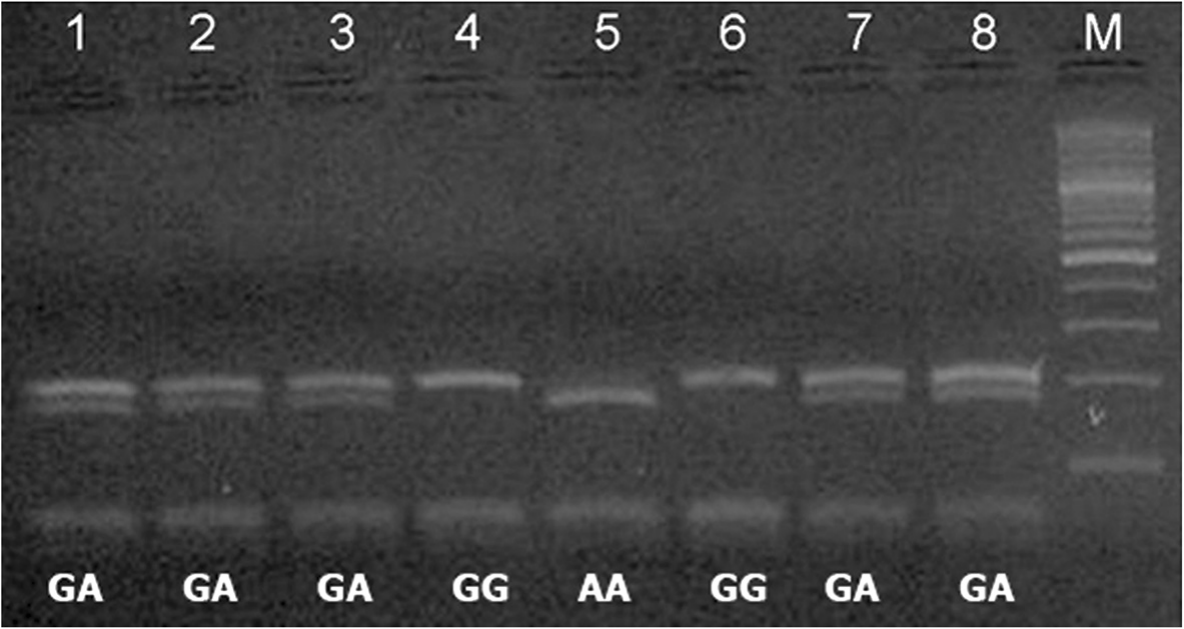
**Genotypes generated from 202 bp PCR product acquired from 8 individuals using spasific primers after digested with Bsn I restriction enzyme.** The lanes 1, 2, 3, 7, 8: GA heterozygote; lanes 4 and 6: GG genotype; lane 5: AA genotype; lane 9: 100 bp marker.

**Figure 2:**
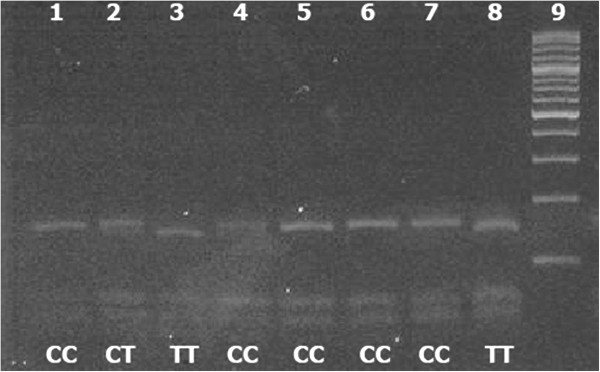
**Genotypes generated from 202 bp PCR product acquired from 8 individuals using spasific primers after digested with Tru I restriction enzyme.** Lane 2: CT heterozygote; lanes 1, 4, 5, 6, 7: CC genotype; lanes 3, 8: TT genotype; lane 9: 100 bp marker.

Photographs of gel products obtained from restriction enzyme digestion with the restriction fragment length polymorphism (RFLP) technique for each polymorphic site are given in Figure 1 and Figure 2. Analyses of the polymorphic sites and of groups with various missing teeth with different combinations were performed. After the PCR and enzyme digestion analysis, the ratio of “A” allele at the −1031 site of the *PAX9* gene promoter region was found to be 34.75% in the hypodontia group, 37.70% in the third molar agenesis group, 31.60% in the hypodontia group except the third molars, and 35.50% in the control group (Table 2). There was no statistically significant difference found between groups. Moreover, the ratio of the G allele was found to be 64.50% in the control group, 65.25% in the hypodontia group, 62.30% in the third molar agenesis group, and 68.40% in the hypodontia group except the third molars (Table 2). There was no statistically significant difference between these groups.

**Table 2:** **Distribution of allels and genotypes of *PAX9 *** **control and test groups**

** SNP**	**Control Group**		**Hypodontia (All)**		**Third Molar Hypodontia**		**Excluding Third molars**	
	**n**	**%**	**n**	**%**	**n**	**%**	**n**	**%**
G-1031A Allel								
A	81	35.5	139	34.75	77	37.7	62	31.6
G	147	64.5	261	65.25	127	62.3	134	68.4
p*			0.913		0.705		0.458	
OR (%95 CI)			1.03		0.908		1.191	
Genotype								
AA	12	10.5	15	7.5	7	6.8	8	8.2
GA	57	50	109	54.5	63	61.7	46	46.9
GG	45	39.5	76	38	32	31.5	44	44.9
p^#^			0.579		0.207		0.676	
Genotype								
AA	12	10,5	15	7.5	7	7.7	8	8.1
GA/GG	102	89.5	185	92.5	85	92.3	90	91.9
p*			0.477		0.633		0.725	
OR (%95 CI)			1.45		1.428		1.323	
GA	57	35.8	109	37	63	42.5	46	33.8
GG/AA	102	64.2	185	63	85	57.5	90	66.2
p*			0.876		0.276		0.809	
OR (%95 CI)			0.948		0.753		1.093	
C-912 T Allels								
T	81	35.5	136	34	79	38.3	58	29.5
C	147	64.2	264	66	127	61.7	138	70.5
p*			0.765		0.611		0.232	
OR (%95 CI)			1.06		0.885		1.311	
Genotype								
TT	14	12.2	19	9.5	11	10.6	8	8.1
CT	53	46.4	98	49	57	55.3	42	42.8
CC	47	41.4	83	41.5	35	34.1	48	49.1
p^#^			0.730		0.426		0.423	
Genotype								
TT	14	12.2	19	9.5	11	10.6	8	8.1
CT/CC	100	87.8	181	90.5	92	89.4	90	91.9
p^#^			0.561		0.876		0.451	
OR (%95 CI)			1.373		1.171		1.575	
CT	53	46.4	98	49	57	55.3	42	42.8
TT/CC	61	53.6	102	51	46	44.7	56	57.2
p*			0.756		0.244		0.695	
OR (%95 CI)			0.904		0.701		1.158	

*SNP* single nucleotide polymorphism, *OR* odds ratio, *CI* confidence ınterval.

*, Chi square test P value.

^#^, Normal SPSS test.

As shown in Table 2, we found no significant difference between the control and test groups in either the −1031 site or the −912 site in terms of gene alleles and their distribution to different groups of agenesis (p > 0.05). Table 3 shows the haplotype frequency of the *PAX9* gene in the control and test groups. The haplotype distribution of both polymorphic sites was consistent with Hardy–Weinberg equilibrium (p > 0.09). When haplotypes as alleles were compared, statistically significant differences were found between the control and test groups (p = 0.05). When compared with other groups, the ratio of individuals having the AC haplotype was found to be less than control groups in congenitally tooth agenesis cases (OR = 3.88; CI = 95%; p = 0.001). It was noticed that in the test group, the individuals having the GT haplotype were less than any other group (OR = 0.67; CI = 0.458–0.993%; p = 0.045). No differences between control and test groups were found when haplotypes were organized as genotypes (p = 0.622). It was noticed that the AC/GC haplotype group was found three times more often in the test group than the control group, but this was not significant (p > 0.05).

**Table 3:** **Haplotype frequency of *PAX9 *** **gene in control and test groups**

**Haplotype G-1031A/C-912 T**	**Control Group**		**Test Group**		**p-Value**
	**n**	**%**	**n**	**%**	**OR (CI %)**
Allel					
AC	55	54,5	46	45,5	0.05 (CS)
GT	55	34,8	103	65,2	
GC	100	35,7	180	64,3	
AT	67	37,2	113	62,8	
AC	55	31.1	46	10.4	0.001( CS)
GT + GC + AT	122	68.9	396	89.6	3.88 ( OR) CI (2,49 – 6,03)
AC + GC + AT	122	68.9	339	76.6	0.05 (CS)
GT	55	31.1	103	23.4	0.673 ( OR) CI (0,458 – 0,993)
Genotype					
GT/GT	0	0	0	0	0,622 (CS)
AT/GC	53	43.4	94	43.1	
AC/AC	0	0	1	0.4	
AC/GC	2	1.6	10	4.5	
AT/GT	2	1.6	5	2.3	
AC/GT	53	43.4	94	43.1	
AT/AC	0	0	0	0	
AT/AT	12	9.9	14	6.4	
AC/GT	53	43,4	94	43,1	0.926 (CS)
GT/GT + AT/GC + AC/GC + AT/GT + AC/GT + AT/AC + AT/AT	69	56,6	125	56,9	

*OR* odds ratio, *CI* confidence interval, *CS* chi-square.

A comparison of the genotype variations of polymorphic sites with various congenitally missing teeth groups is shown in Table 4. Statistical analysis showed that there was no relationship between tooth agenesis groups and genotypic polymorphism variations (p = 0.171). The G-1031A polymorphism and C-912 T polymorphisms were then compared separately with tooth agenesis groups, and the results of this comparison are shown in Table 5. We found no significant relationship between the C-912 T polymorphism and the tooth agenesis group (p = 0.158); a correlation was detected between the G-1031A polymorphism and congenitally missing teeth (p = 0.026) (Table 5).

**Table 4:** **The comparison of the genotype variations of G-1031A and T-912C polymorphic sites of *PAX9 *** **promoter region with various congenitally missing teeth groups**

	**Congenitally missing teeth groups**							**Total**
	**12-22**	**14-24**	**15-25**	**35-45**	**31-32- 41-42**	**18-28- 38-48**	**other**	
GGCC	57	26	12	41	26	82	52	296
	19,3%	8,8%	4,1%	13,9%	8,8%	27,7%	17,6%	100%
GGCT	6	1	0	0	0	1	0	8
	75%	12,5%	0%	0%	0%	12,5%	0%	100%
GGTT	0	0	0	0	0	0	0	0
	0%	0%	0%	0%	0%	0%	0%	100%
GACC	6	2	1	10	2	11	0	32
	18,8%	6,3%	3,1%	31,3%	6,3%	34,4%	0%	100%
GACT	56	18	13	50	40	124	33	334
	16,8%	5,4%	3,9%	15%	12%	37,1%	9,9%	100%
GATT	4	3	2	4	4	11	4	32
	12,5%	9,4%	6,3%	12,5%	12,5%	34,4%	12,5%	100%
AACC	2	0	0	0	0	0	0	2
	100%	0%	0%	0%	0%	0%	0%	100%
AACT	0	0	0	0	0	0	0	0
	0%	0%	0%	0%	0%	0%	0%	100%
AATT	7	3	2	10	5	22	8	57
	12,3%	5,3%	3,5%	17,5%	8,8%	38,6%	14%	100%
TOTAL	138	53	30	115	77	251	97	761
	18,1%	7%	3,9%	15,1%	10,1%	33%	12,7%	100%
							P = 0,171 (X^2^ test)	

**Table 5:** The relationship between polymorphisms on G-1031A and C-912 T regions and missing teeth groups separately

	**Congenitally missing teeth groups**							**Total**
	**12-22**	**14-24**	**15-25**	**35-45**	**31-32-41-42**	**18-28-38-48**	**other**	
GG	63	27	12	41	26	84	52	305
	20,7%	8,9%	3,9%	13,4%	8,5%	27,5%	17%	100%
GA	66	23	16	64	36	165	37	407
	16,2%	5,7%	3,9%	15,7%	8,8%	40,5%	9,1%	100%
AA	9	3	2	10	5	22	8	59
	15,3%	5,1%	3,4%	16,9%	8,5%	37,3%	13,6%	100%
TOTAL	138	53	30	115	67	271	97	771
	18,1%	17,9%	6,9%	3,9%	14,9%	8,7%	35,1%	100%
							P = 0,026	
CC	63	28	13	51	28	93	52	328
	%19,2	%8,5	%4	%15,5	%8,5	%28,4	%15,9	%100
CT	62	19	13	50	40	1134	33	351
	%17,7	%5,4	%3,7	%14,2	%11,4	%38,2	%9,4	%100
TT	11	6	4	14	9	33	12	89
	%12,4	%6,7	%4,5	%15,7	%10,1	%37,1	%13,5	%100
TOTAL	138	53	30	115	67	271	97	771
	%17,7	%6,9	%3,9	%15	%10	%33,9	%12,6	%100
							P = 0,158	

We examined two mutagenic regions (polymorphic sites) of the *PAX9* gene, which were responsible for activating the protein synthesis process. The first mutation described in the human *PAX9* gene was a guanine insertion to the 219^th^ nucleotide in the second exon of chromosome 14 of an oligodontia family [25]. Since this finding, many mutations and one locus deletion of the *PAX9* gene have been reported [12,22,26]. Most of the mutations are located in the paired domain of the second exon.

In our study, two polymorphic regions were examined. No differences were found between the control and hypodontia groups for the A or G alleles (p > 0.05) (Table 2). Our findings are not consistent with the findings of Peres and co-workers [22] as their outcomes showed a high frequency of guanine and thymine alleles in the hypodontia group. Following this, GG (1031) and TT (912) homozygous genotypes were compared between the control and test groups, and no differences were encountered (Table 2). These results are consistent with the findings of Pan et al. [23] but in contrast with Peres et al’s. The reason for this may be due to the adjacency of the Turkish and Chinese populations in terms of geographical origins [27] and that the Brazilian population originated maternally from Amerindians and paternally from Portuguese [28].

Our study comprised third molar agenesis cases, and we consequently found no significant difference between the control and test groups on the basis of alleles or genotypes. In contrast, Peres and colleagues [22] found significant relationships for these criteria. In addition, they also investigated the third molar excluded groups and found that these agenesis types were related to heterozygosis phenotypes. In fact, human family analysis revealed that *PAX9* mutations have a strong connection with third molar agenesis, indicating that *PAX9* mutations have a lesser but distinct effect on incisors and premolars.

In our study, the haplotype frequencies of genotypes and their relationship with the patient and control groups were also examined. GT, GC, and AT alleles seen in cases of patients with congenital missing teeth were found to be a significant risk. In other words, the individuals having an AC allele were found to have a lower risk of facing tooth agenesis (p ≤ 0.05). We conclude that no relationship exists between the AC haplotype and tooth agenesis. In parallel with this finding, when comparing the AC allele with GT, GC, and AT alleles, the risk of missing teeth in individuals having an AC allele is 3.88 times lower than individuals having other alleles (OR = 3.88; CI = 2.49–6.03%; p ≤ 0.001). The ratio of the GT haplotype in the control groups was significant. It is possible that subjects having this allele have a low risk of tooth agenesis (OR = 0.674; CI = 0.458–0.993%; p = 0.045). The findings of Peres et al. were consistent with the present study. After examining the two related polymorphic sites, compared to the GA and AA genotypes, the GG genotype was seen less in the third molar missing group but there was higher frequency in groups missing lower and upper canines, upper central incisors, and lower and upper second molars (p = 0.026). Thus, we conclude that individuals with a GG genotype are unlikely to show a lack of congenital third molars and are likely to show a lack of canines, first and second molars, and upper central incisors. However, it is not possible to infer this for T-912C (p > 0.05).

The discrepancy of the results between the studies may be explained as follows. As demonstrated in our study, genetic polymorphisms often show ethnic variation. For example, in the study of Peres and colleagues on the G-1031A polymorphism, the G allele frequency was 25%, but in our study this frequency was 65.25%. According to the study of Pan et al. (2008), this frequency value was 48%. Thus, further studies of different ethnic populations are needed to illuminate the exact relationship between the *PAX9* gene polymorphism and congenital tooth agenesis. The second reason is that the diversity of missing teeth phenotypes of patients may give rise to different outcomes. For example 70% of patients lack the third molar in the study by Peres (2005), whereas this percentage was 55% in our study. Pan (2008) had excluded the third molars. We suggest that this diversity may disappear if future studies are based on genotypes of different populations with the same missing teeth groups. Past studies have contributed a great deal to our knowledge about the process of congenital missing teeth. However, the molecular mechanism underlying this abnormality is still not fully described. The mismatch between high incidences of tooth agenesis with low-value findings of agenesis studies [15,29,30] suggest that congenital lack of teeth may be a more heterogeneous structure than expected and several independent defective genes working in parallel or in combination with other genes can lead to the formation of specific phenotypes.

For the Turkish population, limited number of studies investigating the genetic etiology of congenital missing teeth anomaly was found. To understand the molecular basis of tooth agenesis, to investigate the variations of different populations, and to explain the relationship between genetic variants and variable phenotypes clearly, further studies should analyze both the *PAX9* gene and the other candidate genes, by increasing the sample size and using the latest laboratory techniques that are proven to give more definitive results. In order to develop the curing techniques and connect these results with contemporary medical treatment alternatives in the future, investigators dealing with tooth agenesis may think of gathering samples from oral tissues and build the studies up to the tissue-like structures and engineering methods which might be one of the highest limitations of these kinds of studies in addition with gathering ethical approval for both animal and human studies.

## Conclusions

Our study revealed that (1) in the *PAX9* gene, no significant relationship between the hypodontia group and the control group was found in terms of both the A or G allele in the G-1031A polymorphic site; (2) the risk of missing teeth in individuals having the AC allele is 3.88 times lower than individuals with other alleles; (3) individuals having the GG genotype have a low risk of third molar agenesis; (4) the subjects having the GT allele can be assumed to have a low risk of tooth agenesis; (5) polymorphisms in the promoter region of the *PAX9* gene may have an influence on hypodontia in humans.

## Methods

### Patient selection and control group

Inclusion criteria were as follows:

1. All patients having hypodontia were between 12 and 32 years of age and every non-missing permanent tooth was apparent in panoramic X-rays, including third molars and/or their germs.

2. Patients and families were off systemic diseases and hereditary abnormalities.

3. Spaces in the arches of patients were only from deciduous teeth.

4. Patients having doubts about the extraction history were excluded.

Two hundred patients meeting these criteria were chosen for the study group. The control group was organized according to these criteria:

1. Subjects must have proper tooth number and shape.

2. Thirty-two teeth must be properly seen in panoramic radiographs.

3. Subjects must be genetically and hereditarily healthy.

One hundred and fourteen controls were included. All received detailed clinical, intra/extra oral, radiological examination. General characteristics including age, gender, ethnicity, health status, birth defects, and family history, were documented. Subjects declared that their ancestors were Turkish. Two milliliters of venous blood samples were collected in *EDTA* (Ethylenediaminetetraacetic acid - an anticoagulant for blood samples-) tubes from these 314 subjects.

### Ethical approval

The study design was approved by Selcuk University Faculty of Dentistry Ethical Committee (Approval Number: 228; 2007,13–1). Informed consent was prepared and signed by every patient participating in the study or in the case of children, by the parents or legal guardians.

### DNA isolation and polymerase chain reaction (PCR)

DNA was extracted using a special isolation kit (UltraClean^TM^ DNA BloodSpin Kit; Mobio Lab Inc., Carlsbad, CA, USA). A microcentrifuge (13,000 g) (Eppendorf North America, Inc. Westbury, NY, USA), water incubator (65°C), micropipette (20 ml and 500 ml volume), and a vortex were also used throughout the process.

All the isolation steps were carefully performed according to the manufacturer’s instructions. After the last step, genomic DNAs were obtained at the bottom of the tubes and were stored at −20°C. Polymorphisms located in the 1031 and 912 regions were detected and reproduced as described in Peres et al’s study [22].

PCR tubes contained 10 μM Tris–HCl (pH 8.3), 50 μM KCl, 1 μM of each primer, 200 μM of each dATP, dCTP, dGTP, dTTP, 2.5 μM MgCl2, and 2.5 U Tag DNA polymerase (Amerham Pharmacia Biotech, Uppsala, Sweden).

The incubation process of the thermo cycle of the PCR (Geneamp PCR systems 9700; Applied Biosystems, Perkin – Elmer, Foster City, CA, USA) was performed as follows: 5 min at 95°C, 10 cycles of the triple steps that are 30 s at 95°C, 30 s at 60°C, and 30 s at 72°C; then another 10 cycles of the triple steps that are 30 s at 95°C, 30 s at 58°C, and 30 s at 72°C; and finally 5 min at 72°C.

### Gel electrophoresis

2% agarose (A5093, Sigma, St. Louis, MO, USA) was mixed with TAE (Tris, acetic acid, and EDTA) solution and boiled for 1 min to prepare agarose gel. 5 μl of the PCR product was mixed with the loading dye and inserted into the pits. The mixture was processed through gel electrophoresis (Horizon 11–14; Life Technologies Inc., Paisley, Scotland) for 45 minutes under 120 V. 100-bp DNA ladder was used in the first pit of the gel. The 202-bp PCR product was observed under a ultra-violet (UV) illuminator (TFX 20 M-Vilber Lourmat; Mar-na La Vallee, France) and recorded.

### Restriction enzyme digestion and genotyping

The rest of the 15-μl PCR product was used for restriction enzyme digestion. A 6-μl mixture was prepared consisting of 3.5 μl sterile dH_2_O, 2 μl of restriction enzyme buffer, and 0.5 μl restriction enzymes (*HaeIII* and *MseI*). This mixture was added into the PCR product and rested in a water bath at 37°C for 12–16 h.

A 3% agarose gel was prepared again, and the obtained digestion product was loaded into this gel. A 100-bp DNA ladder was added, and after 45 min at 120 V, the bands were observed under a UV illuminator (TFX 20 M-Vilber Lourmat; Mar-na la Vallee, France).

### Statistical analysis

Acquired data were statistically analyzed using the program SPSS (version 15.0). The chi-square test was used, and the power of significance of the data was tested. Inter-group relations were evaluated using an odds ratio (OR), and the results were arranged and shown in tables. The significance level was given as 0.05.

## Competing interests

There are no financial or non-financial competing interests (political, personal, religious, ideological, academic, intellectual, commercial or any other) to declare in relation to this manuscript.

## Authors’ contributions

EI did the decision making and worked in clinical section. He examined the patients, collected bloods and stored them. SN took the bloods and participated in the genetical laboratory part. HA designed the primers and helped in connecting with companies. ZS helped Dr. EI to analyze the outcomes statistically and to write the manuscript. All authors read and approved the final manuscript.

## Authors’ information

Dr. Eren Isman is the head of the department of Orthodontics, Gaziantep University, Gaziantep, Turkey. Dr. Suleyman Nergis is working as a post-doctor in Medical genetics department in Selcuk University, Konya, Turkey. Dr. Hasan Acar is the head of Medical Genetics Department in the same faculty as Dr. Suleyman. Dr. Zafer Sari is the dean and Orthodontic department head of Akdeniz University, Faculty of Dentistry, Antalya, Turkey.
